# An immune epigenetic insight to COVID-19 infection

**DOI:** 10.2217/epi-2020-0349

**Published:** 2021-03-09

**Authors:** Bimal P Jit, Sahar Qazi, Rakesh Arya, Ankit Srivastava, Nimesh Gupta, Ashok Sharma

**Affiliations:** ^1^Department of Biochemistry, All India Institute of Medical Sciences, New Delhi 110029, India; ^2^Regional Institute of Ophthalmology, Institute of Medical Sciences, Banaras Hindu University, Varanasi 220115, India; ^3^National Institute of Immunology, Aruna Asaf Ali Marg, New Delhi 110067, India

**Keywords:** ACE2, COVID-19, DNA methylation, histone PTMs, host-virus interaction, immune evasion, immunoepigenetics, SARS-CoV-2

## Abstract

Severe acute respiratory syndrome coronavirus-2 is a positive-sense RNA virus, a causal agent of ongoing COVID-19 pandemic. *ACE2R* methylation across three CpG sites (cg04013915, cg08559914, cg03536816) determines the host cell’s entry. It regulates ACE2 expression by controlling the SIRT1 and KDM5B activity. Further, it regulates Type I and III IFN response by modulating H3K27me3 and H3K4me3 histone mark. SARS-CoV-2 protein with bromodomain and protein E mimics bromodomain histones and evades from host immune response. The 2′-O MTases mimics the host’s cap1 structure and plays a vital role in immune evasion through Hsp90-mediated epigenetic process to hijack the infected cells. Although the current review highlighted the critical epigenetic events associated with SARS-CoV-2 immune evasion, the detailed mechanism is yet to be elucidated.

The advent of budding infectious agents like COVID-19 has resulted in a remarkable impact on the discrepancy of human resources, lifestyle, economy, and longevity in the modern era. COVID-19 infections have been associated with respiratory dysfunction that contributed to substantial alterations in the clinical manifestation and has become a significant public health concern [1]. The severe acute respiratory syndrome coronavirus (SARS-CoV-2), β-enveloped, segmented, positive-sense RNA belong to Orthocoronavirinae subfamily. Mostly attacks mammals and could cause potentially fatal respiratory dysfunctions. The previous form regarded as 2019-nCoV, the COVID-19 has transmitted rapidly throughout China and received tremendous attention worldwide [2]. However, the SARS-CoV incidence was in 2002 followed by Middle East respiratory syndrome coronavirus (MERS-CoV) in 2012, followed by the subsequent entry of highly pathogenic large-scale epidemic coronavirus, COVID-19 as observed in the 21st century in the human population. Recent data as of 1 January 2020, indicates, a total of 82,579,768 cases globally, with mortality of 1,811,849 cases affecting 213 countries.

Out of the whole affected population, Bharat (India) accounts for 10,305,778 confirmed cases with a mortality of 149,218 cases [1]. Although the actual evidence regarding the virus’ origin remains unclear, several studies indicate that the bat population in Wuhan city’s seafood market in China is the potential reservoir [3]. Population genetic analysis revealed that the virus’s L-type strain is more aggressive and contagious than the S strain [4]. The genome sequencing approach’s results indicate that COVID-19 shares 96.2% overall genomic similarity with Bat-CoV-RaTG13 suggests a typical ancestral relationship [5]. According to recent Lancet findings, Acute Respiratory Distress Syndrome is the most common pathological event of COVID-19 characterized by cytokine storm, resulting from the massive secretion of pro-inflammatory cytokines and chemokines during the infection by immune effectors cells [6]. Immunocompromised populations associated with several genetic and nongenetic diseases are highly vulnerable to infection and acts as the potential risk factor modulating the clinical severity. Evidence suggests persistent viral attacks associated with altering host epigenetic machinery, thus paving a way to evade and subvert the immune system for a successful infection strategy [7–9]. Therefore, it is highly crucial to understand the host-cell chromatin dynamics to regulate the expression of specific genes adapted by COVID-19-host confrontation during its infection. Here, we provide a brief insight on possible epigenetic changes associated with coronavirus and other viruses to evade host immunity.

## Host epigenetic architecture

**Figure 1.**
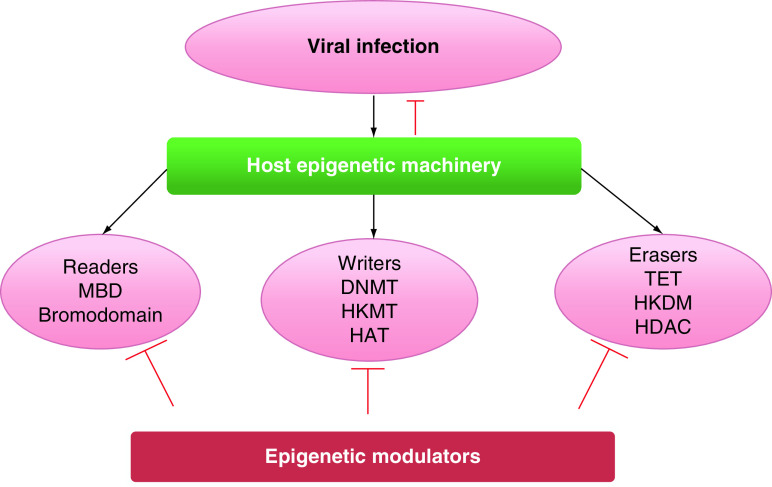
Epigenetic approach to viral infection.

Despite the Mendelian inheritance, which emphasizes the transmission of genes associated with a particular trait, discovery in several high throughput technologies contributed significant advancement to epigenetics. Epigenetics elucidates gene expression regulation and silencing events without any change in the DNA sequence. Although the changes instigated by this are stable, reversible modifications in the DNA sequences could induce a dramatic change in parent and progeny phenotypes. The consequences may persist for a lifelong period affecting cellular behavior [10]. Deciphering the fine-tuning mechanisms associated with an epigenetic signature could lead to identifying a potential target that will be suitable for identifying severity markers of a particular disease. Understanding the host-viral functional network is crucial to control the virus’ pathogenesis and infectivity (Figure 1). More recently, epigenetic has become an emerging field in controlling the host innate and adaptive immune system induced by viral pathogenesis. Being originated by environmental stimuli, the field encompasses two major events methylation and acetylation, which play a crucial role in altering the chromatin packaging and position of regulatory elements like promoter or enhancers. These methylation and acetylation processes are being carried out by DNMTs and HDACs, which have varying expression levels in different cells under different conditions. In addition to this, other histone biochemical modifications includes phosphorylation, sumoylation, ubiquitination. Histone modification enzymes portray their role in coordination with other chromatin regulators like ATP-dependent chromatin remodeling complexes and the contrasting effects of the polycomb group and trithorax group genes [10]. Normal functional cells with transcriptionally active genes characterized by unmethylated promoter CpG islands, histone hyperacetylation, H3 lysine 4 (H3K4) di- and tri-methylation and H3K79 methylation. In addition to this, the transcriptionally repressed genes are discerned with promoter CpG island methylation, histone hypoacetylation and H3K9, H3K27 methylation [11].

## Epigenetic basis of viral immune evasion

Many DNA and RNA viruses harbor epigenetic marks to maintain their persistency and latency. A previous study by Shamay *et al.* [12], revealed LANA expressed by Kaposi’s sarcoma-associated virus (KSHV or HHV8) could interact with cellular DNMTs [13]. Recruitment of DNMT3A by LANA and initiation of DNA methylation in B cell lymphoma have been also reported [14]. Mechanically, LANA interacts with the gene promoter of TGF-β Type II receptor (TGF-βRII) inducing DNA methylation and causes transcriptional silencing [14]. It was also evident that promoters of several IFNγ-regulated genes with STAT1 binding sites are the potential target for KSHV associated with repression of IFNγ-induced gene activation [7]. Interaction of KSHV polyadenylated nuclear RNA with host nuclear regulators, like PRC2 and IRF4, also shown to modulate the expression of several cytokines [14]. In particular, an increase in DNMT is crucial in regulating the viral latency by modifying the epigenetic reprogramming of infected cells. Furthermore, it was elucidated that trimethylation of H3K27 probably through PRC2 is associated with CpG methylation of the tumor suppressor gene, as shown in Epstein–Barr virus (EBV) latency in B cells [15]. As evident, Epstein–Barr-virus encodes a LMP1 and LMP2A, leading to activation of downstream signaling molecules like JNK, AP-1 and STAT3 causing upregulation of the host DNMT1 [16]. Besides, the role of oncoprotein E7 of human papillomavirus in modulating the DNMT1 and H3K27 methyltransferase EZH2 activity in cervical cancer has been reported earlier [8]. Furthermore, the modulation of host epigenome by simian vacuolating virus 40, adenoviruses, HIV and human T cell virus-1, paramecium bursaria chlorella virus, provides significant insight into the virus controls host epigenetic integrity and machinery for its efficient propagation by modulating gene repression [9].

Thus far, the most comprehensive evidence of the modification of chromatin at specific gene locus as observed in some viruses that play a crucial role in regulating host epigenetic and immune evasion. Interference in the Th-1 cell immune response stimulated by the respiratory syncytial virus infection, upregulates H3K4 demethylase KDM5B, results in Type 1 interferon and cytokine responses. As evidence elucidates the association of hepatitis B and C virus with hepatocellular carcinoma and their positive correlation with the host genome’s aberrant DNA methylation [17–19]. DNA methylation-dependent repression of IL-4 receptor was observed during hepatitis B virus infection, mediated by the recruitment of DNMT3A by HBX [20]. Recent results indicate the HIV encoded tat protein regulates RNA polymerase II activity and fine-tune the expression of early response genes and makes the host cellular environment hospitable for the virus [21]. Furthermore, the association of HIV infection with upregulated DNA methylation event at CpG site of FOXOP3 locus leading to decreased production of TGF-β and increased production of interleukin IL-4, thus alters the Treg cell function [22].

Notably, epigenetic processes are incredibly crucial in controlling the viral DNA-based processes like genome replication, DNA damage response, a temporal cascade of transcription provides a footprint for understanding and targeting the viral pathogenesis and infection [23]. Certain viruses develop a common strategy where the early proteins of viruses target the cellular processes by binding the cellular regulatory factors. As observed in DNA tumor viruses; polyomaviruses, adenoviruses and papillomaviruses propagate their productive infection and cell transformation by binding to the p53 gene and regulating the cell cycle [24]. For instance, adenovirus e1a protein binds the host acetyltransferase, p300/CBP promoting viral replication by disrupting histone acetylation. Late-expressed protein VII binds host chromatin and sequesters the danger signaling HMGB proteins in chromatin resulting in downregulation of HMGB-induced immune response [25]. Further evidence showed that e1a blocks hBRe1 ubiquitin ligase complex formation required for ISGs in response to adenovirus infection [26].

Besides this, miRNA’s role derived from RNA and DNA viruses plays a prominent role in immune evasion. miRNA derived from RNA viruses have been shown to play a significant role in controlling host immunity. miRNA from several human herpesviruses, polyomavirus JC and murine cytomegalovirus provide subtle evidence of miRNA’s potential role in host immune evasion [27,28]. Ebola virus (EBOV/EBV) miRNA overexpression (EBOV-miR-1-5p) causes inhibition of importin-alpha5, leading to immune evasion from the host interferon response [29]. SV-40 encoded miRNA also regulates the viral gene expression and decreases the susceptibility to regulatory T cells [30]. Viral miRNA derived from the KSHV, herpes simplex virus 1, human cytomegalovirus shown to modulate the host innate and adaptive immune response [31]. Evasion from the adaptive response was observed from the miRNA derived from SV40 and murine polyomavirus by negatively regulating the early gene expression [32]. Similar results were observed in the KSHV and EBV virus [33]. Furthermore, miR-146a dampen the host IFN-β production-mediated by TRAF6, thus increasing the replication and infectivity ability of dengue virus [34].

Whether in cell culture or mouse model, epigenetic drugs are currently the potential candidates that act as prominent antiviral drugs. More recently, the HDAC inhibitors like panobinostat (NCT01680094), vorinostat (NCT01319383) and romidepsin (NCT02092116, NCT01933594), VPA (NCT00289952) are used in combination with antiretroviral therapy. However, this attribute is limited by the incomplete latency reversal or insufficient clearance of latency-reactivated cells, which further seeks immune enhancement treatments [35,36]. Interestingly, some HDAC inhibitors like arginine butyrate and ganciclovir could induce lytic phase gene expression that acts as a potent sensitizer observed in EBV-associated lymphoma [36,37]. DNMT inhibitor azacytidine has been observed to reverse the dense CpG methylation and induces gene re-expression in patients with EBV tumors [38]. The previous study has explained the potential role of DNMT2 in retrotransposon silencing, and the results revealed overexpression of DNMT2 altered several genes associated with viral infection [39].

The cross-talk between host and viral infection could be exemplified by the potential miRNA specific to the viral infection that could be used for the post-transcriptional regulation of target gene expression. Upadhya *et al.*, 2014 highlighted the association of CpG methylation as a genomic signature for large DNA viruses infecting invertebrates [40]. Further study also revealed the promoter hypermethylation as possible indicators of human papillomavirus-infected patients with head and neck squamous carcinomas [41]. Significant correlation of EBV oncoprotein EBNA3C and its positive association with transcriptional activation of autophagy genes by recruiting active epigenetic marks like H3K4me1, H3K4me3, H3K9ac and H3K27ac has been elucidated previously [42]. Furthermore, the prospective regulatory role of hsa-miR-374b-5p miRNA on Type 1 interferon expression in Japanese encephalitis virus-infected microglial cells was also reported earlier [43–45]. Immune metabolic findings show that viral protein ICP0 is an attenuator of TLR signaling inhibiting innate response to herpes simplex virus [46].

## Epigenetic approach of coronavirus immune evasion

### Epigenetic alterations of *ACE2R* determines SARS-CoV entry into the host cell

Maintaining a latency stage inside the host, mimicking the host immune system requires manipulation of chromatin and heterochromatin assembly by viruses. It has become clear that the coronavirus’ spike protein facilitates its entry into the target cells mostly by the surface unit s1 of S protein upon S protein priming. The molecular affinity between the ACE2 receptor, mostly expressed on the Type–II lung epithelial cells, is crucial in the viral entry [47–50]. Spike protein and receptor affinity is a crucial determinant of tissue tropism, which plays a vital role in the disease’s etiopathogenesis. Therefore, it is crucial to understand the epigenetic signature of the *ACE2* gene for controlling the initial step of entry and fusion of the virus.

The genome-wide DNA methylation array and chip methylation pipelines study indicates the varied degree of DNA methylation of the *ACE2* gene in different tissue subtypes. The lowest *ACE2* gene methylation across three CpG sites (cg04013915, cg08559914, cg03536816) was predominant in lung epithelial cells compared with other tissues [51]. A subsequent study shows that *ACE2* gene hypo-methylations are mostly confined to the females compared with the male, suggesting angiotensin II metabolism and its association with hormonal differences or genetic differences in chromosome dosage [52]. Moreover, transcriptomic analysis shows a lack of possible association of ACE2 with race, age and gender. However, Asian smoker population exhibits higher ACE2 than the nonsmokers suggesting an epigenetic impact on ACE2 activity in the respiratory system [53–55]. However, the validation of the approach necessitates proteomic data. Previous evidence also highlighted hypo-methylation mediated overexpression of ACE2 and its association with the onset of severity in the patients of systemic lupus erythematosus, an autoimmune disease upon infection of SARS-CoV-2 with peripheral blood T cells [56]. Inconsistent with this, further study shows the prospective role of TNF-α in the regulation of ACE gene transcription and pathological complexity in endothelial cells. The results indicate that TNF-α enhances DNA methylation in the ACE promoter by decreasing the activity of DNMTs, DNMT3a and DNMT3b and TET1 [57]. Transcriptomic and system biology approach revealed the significant association of higher expression of ACE2 with RAB1A, HAT1, HDAC2 and KDM5B in patients with other comorbidities like hypertension, diabetes and chronic obstructive lung disease [58]. Also, the role of NAD+ dependent histone deacetylase, SIRT1 in the induction of ACE2 activity by stimulating the ACE2 promoter was reported during energy stress indicates SIRT1 could be a target for epigenetic drugs in context to COVID-19 infection [54]. Pathway enrichment analysis also revealed the potential role of KDM5B in regulating the expression of several genes associated with ACE2 possibly by acetylation and methylation epigenetic marks like H3K4me1 and H3K4me3, as well as H3K27ac [56]. Although the literature is scanty about the COVID-19 ACE2 epigenetic pattern, the molecular mechanism regulating the ACE2 activity cannot be undermined so far as the current pandemics and pathogenesis are concerned. A recent study has observed that SARS-CoV-2 cross-reactive CD4^+^ T cells are still present in 70% convalescent patients even after 5 months of the infection [57].

### Coronavirus induces epigenetic modulation of immune cells, alters antigen presentation & interferon response

It is apparent that the generation of useful anti-inflammatory cytokines and chemokines inhibit viral replication and enhances antigen presentation [57–59]. The ISG response plays a prominent role in controlling the viral infection for efficient immune function. Type 1 IFN induces a cascade of signaling events which causes transcription of several ISG [60,61]. Although evidence is scanty from SARS-CoV-2 in this perspective, studies from influenza and other RNA respiratory viruses provide significant insight. SARS-CoV-2 and MERS-CoV viruses are found to delay ISG expression significantly. Transcriptomics and proteomics findings in Calu3 cells revealed diverse virus-specific ISG expression signatures. SARS-CoV-2 infection of Calu3 cells revealed a strong induction of ISG effectors, but the response was significantly delayed with peak expression at 48 h post-infection. In 2012, the newly emerged MERS-CoV showed a dramatic delayed ISG released with effects visible at 18 h post-infection, for the decreased expression of potential ISG subsets [62].

Further evidence supports the notion that downregulation of ISGs is not due to any impairment in the signaling cascade, but by histone modification like methylation and acetylation induced by a pathogen. Upon viral infection like SARS-CoV-2, the host produces Type I and III IFN, which induces histone modulation complex, which renders removal of repressive histone mark (H3K27me3) inducing activating mark like H3K4me3. This conversion of inactive chromatin to active chromatin allows the binding of several transcription factors like STAT1 and IRF7 thus inducing the ISG expression [63,64]. However, incorporating repressive histone modifications like H3K27me3 and removing active mark H3K4me3 could impose a more condensed state of chromatin which prevents binding of transcription factor and thus reduces ISG expression. Additionally, inhibition or downregulation of an H3K79 methylase, Dot1L enzyme associated with decreased antiviral response and facilitates viral replication, suggests its crucial role in antiviral response [65].

The previous study’s prospective interleukin role in regulating the epigenetic signature has been provided [66]. The author revealed that treatment with IL-1 induces STAT-6 activity; a major transcription factor for IL-4 mediated signaling that binds to the H3K27 demethylase Jmjd3 promoter. An elevated level of Jmjd3 decreases H3K27 dimethylation and trimethylation (H3K27me2/3), leading to transcriptional activation of the M2 marker gene. The significant association of H3K9me2 as a suppressor of IFN inducible antiviral response has been elucidated by Fang *et al.* (2012) [67,68]. However, inactivation of lysine methyltransferase G9a an inducer of H3K9me2 resulted in high IFN production, suggesting that methyltransferase could be an ideal therapeutic epigenetic target challenge the viral invasion. Interestingly, results of Menachery *et al.* (2018) revealed a possible association of H3K27me3 global methylation with downregulation of ISG and DNA methylation of antigen presentation gene upon MERS-CoV and H5N1VN1203 [66]. The possible association of TNF-α and H3K4me3 in the induction of trained innate immunity in monocytes and DC1 antigen presentation and TH1/TH17 immunity upon infection has been described previously [67,69]. Mechanistically, it was observed that an increase in either TNF-α or IFN-γ is sufficient to induce the MLL1 activity which stimulates H3K4 methylation and is required for DC stabilization [67]. Further evidence by Liu *et al.* supported the potential role of influenza virus NS1 in modulating the JAK-STAT signaling by facilitating the export of DNMT3b from the nucleus to the cytoplasm and its subsequent degradation by K48-linked polyubiquitination. Promoter demethylation leads to the expression of specific JAK-STAT signaling suppressors such as SOCS1, SOCS3, PIAS1 and induces inhibition of interferon signaling in an autocrine or paracrine manner [68].

**Figure 2.**
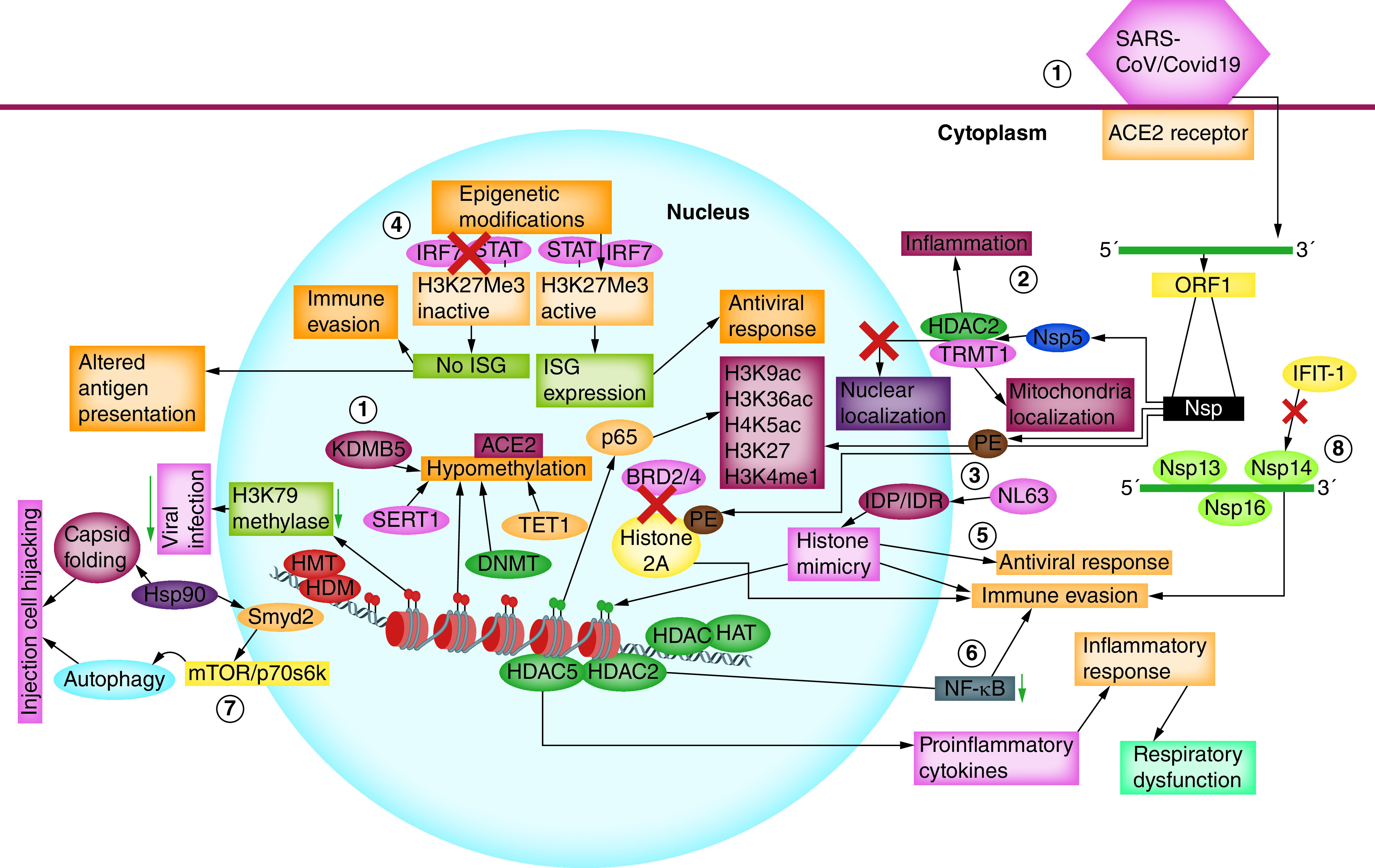
Interaction of coronavirus and modulation of host epigenetic machinery. **(1)** *ACE2R* hypomethylation across CpG sites in lung epithelial cells/other cell types is the first step for entering the coronavirus. DNA methylation in the ACE promoter regulated is under the strict regulation by DNMTs, DNMT3a and DNMT3b, TET1, MAX, KDM5B, HDAC2. **(2)** Virus fuses taking advantage of host cell membrane endocytic pathway. Upon the SARS-CoV entry, the positive strand RNA releases to the cytoplasm and serves as a template for negative strand, which subsequently produces more positive stand and mRNA leading to viral protein synthesis. The Nsp5 protein interacts with both TRMT1 and HDAC2 and prevents their nuclear entry. **(3)** The protein E of SARS-CoV-2 shares similarity with histone 2A and prevents its interaction with BRD2 leading to evasion from host defense. **(4)** Infection with SARS-CoV-2, host, produces Type I and III IFN by removing the repressive histone mark (H3K27me3) and activating H3K4me3, induces chromatin in an open state and permits the binding of transcription factors like STAT1, IRF7 leading ISG expression. However, the incorporation of repressive histone marks like H3K27me3 could impose a more condensed state of chromatin which prevents binding of transcription factor and thus reduces ISG expression inducing immune evasion. **(5)** IDP or IDR derived from SARS-CoV-2 or NL63 coronavirus interacts with host SLiMs proteins that share certain residues of histone proteins play an important role in modulating gene expression histone mimicry. SLiMs are also a potent target for binding of epigenetic readers. This contributes to viral pathogenicity and suppresses antiviral response. **(6)** Nuclear localization of HDAC5 a Type II HDAC induces pro-inflammatory cytokines and controls inflammatory response. In response to SARS-CoV infection, HDAC2 activity increases, leading to NF-κB activity inhibition, altering monocyte and macrophage function and modulating host cell response. **(7)** SARS-CoV-2 uses Hsp90, which hijacks infected cells and causes autophagy by regulating SMYD2 and mTOR/p70S6K signaling. **(8)** Ifit1, an interferon-induced RNA binding protein, binds to viral RNA lacking 2′-O-methylation at their 5′ end and preventing RNA translation. ORF1 of viral RNA translated into polyproteins consists of Nsp13, Nsp14, Nsp16. Nsp16 possess methyltransferase (2′-O-MT) activity, nsp13 function as 5′ triphosphatase and helicase activity, nsp14 exhibits N7MT activity. Viral Nsp16 with 2′-O MTases and mimics c. IDR: Intrinsically disordered region; SARS-CoV: Severe acute respiratory syndrome coronavirus.

Acetylation and deacetylation of histones play a significant role in macrophage activation and survival. HDAC mediates the regulation of non-histone proteins involved in mechanisms crucial for cellular functions like DNA repair, replication, P53 signaling, HIF-1α, STAT3 or p65. HDAC macrophages display a pro-inflammatory function by producing pro-inflammatory cytokines like TNF-α, MCP-1, IL-1α, IL-1β and IFN-γ and are a potential target for HDAC inhibitors [69]. The role of HDAC2 in modulating the NF-κB activity plays a significant role in the immune evasion strategy of SARS-CoV-2. Nuclear localization of HDAC2 makes it convenient to inhibit the NF-κB activity, thus altering the monocyte and macrophage function [70]. Overexpression and knockdown study with HDAC5, a Type II HDAC indicates the subsequent activation of TNF-α and MCP-1 in macrophages contributing to the inflammatory response. Respiratory dysfunctions associated with COVID-19 infection are exacerbated by inflammatory response stimulated by monocyte and macrophages. Monocytes play a crucial role in innate immune response, which migrated to the affected areas and differentiated into macrophage and plays a defensive role by producing pro-inflammatory cytokines like IL-1β, TNF-α, IL-6 and chemokines that facilitate migration [71]. HDAC5 localized in the nucleus and participated in the inflammatory response. However, drugs that could activate the HDAC2 and facilitate nuclear export of HDAC5 could be a novel strategy to control the inflammatory response associated with COVID-19. Current evidence indicates methylxanthine theophylline, macrolide antibiotics, the tricyclic antidepressant nortriptyline, the volatile anaesthetics isoflurane, the phenolic compounds gallic acid and curcumin as well as the plant bioactive molecule andrographolide have the potential to induce the HDAC2 activity by inhibiting PI3K-δ signaling (Figure 2) [72–75].

### SARS-CoV-2 protein 3b as an epigenetic modulator

Characterization of viral protein components and their host interacting partners could provide novel insight to understand the molecular basis of immunomodulation strategy. Recent evidence indicates the potential role of SARS-CoV-2 protein 3b an accessory protein component, has been observed to interacts with the host protein machinery like RUNX1b. Given that RUNX1b stimulates transcription of genes involved in definitive hematopoiesis and T cell differentiation cytokines and chemokines including IL-2, IL-3, GM-CSF, MIP-1α, CSFR, etc. [74,75]. In response to SARS-CoV-2 infection protein, 3b stimulates phosphorylation of RUNX1b in an ERK-dependent manner and activates IL-2 promoter [74]. This high degree of molecular linkage of RUNX1 is thought to be mediated by HDAC recruitment and T cell cytotoxic response [76]. The evidence supports the notion that efficient T cell function or exaggerated immune response might be epigenetically controlled.

### Histone mimicry as a basis for modulation of gene expression & immune evasion

Coronavirus is enveloped single-stranded positive RNA viruses with a genome size ranging from 26.2 to 31.7 kb. The large capped polyadenylated genome constitutes a set of conserved genes arranged in a particular order: 5′ORF1a-ORF1b-S-ORF3-E-M-N-3′. Among these ORF; ORF1a/b exhibits two-thirds of the genome and produces an mRNA (mRNA1) which encodes different structural and functional protein components. The structural proteins include: S, E, M and N. The N proteins of CoV-2 consist of three conserved domains: N terminal domain (NTD), C terminal domain (CTD) separated by intrinsically disordered regions (IDR) or RNA binding domains. It was observed that the NTD preferentially binds the 3′ end of viral RNA by electrostatic interaction which stabilizes RNA structure and acts as a chaperone and helps in replication. The CTD plays a vital role in the dimer-dimer association, protein interaction and stress response [77]. The NTD and CTD are separated by IDR or IDP which lack 3D shape in their native conformation. These domains play a crucial role in DNA, RNA and protein binding and enhance the RNA binding activity of NTD and CTD. It was also pointed out that these IDPs play a predominant role in viral adaptation, evasion from the host immune system. Regulation of viral protein synthesis managing the economical use of genetic material via alternative splicing, overlapping genes and antisense transcription [78]. It is surprising to note that the human coronavirus NL63 has 7.3% of these disordered residues [79]. The IDP or IDR are also observed in the mammalian proteome characterized by a predominant hydrophilic amino acid with a low abundance of bulky hydrophobic amino acids. Post-translational modification and sharing of the particular motif of eukaryotic proteins like SLiMs make them ideal for regulating the host defensive strategy. Short linear motifs commonly known as SLiMs, also the components of eukaryotic linear motifs. SLiMs are observed to be a part of histone proteins and acts as a binding target for readers. As the binding affinity and specificity of SLiMs reside in 2–5 residues, it makes it easy to mimic the host SLiMs. It was observed that the presence of histone H3 like sequence within the C-terminal portion of NS1 of the H3N2 subtype of influenza virus [80].

The role of NS1 protein in suppressing Type I IFN response during infection has been described previously. NS1 protein consists of a sequence of 226-ARSK-229 which resembles the first four amino acids (1-ARTK-4) of histone H3. The tails of influenza may contain PDZ ligand (PL) motifs whereas, SUMOylation sequence in the NS1 tail of H1N1 strain [81]. The PL motif observed in ESEV and EPEV within the NS1 tails of avian-derived influenza strain are significantly associated with pathogenicity and could suppress the antiviral response. The PL motif can attenuate the apoptosis of infected cells and increases the viral load [82].

Furthermore, the histone mimicking ability of NS1 of influenza virus and its modulation of host epigenome has been well explained [80]. The study revealed carboxy-terminus of the H3N2 protein NS1 and tail of histone H3 shares homologs sequence. The NS1 protein interacts with the human PAF1 transcription elongation complex (hPAF1C) and decreases the PAF1-mediated antiviral response in a host. Furthermore, the binding affinity of histone H3 tail and H3N2 NS1 tail to PAF1 has been explained previously, contributing to RNA elongation co-transcriptional process [80]. Interaction between CHD1 and PAF1 in the regulation of transcriptional elongation has been elucidated previously. WDR5 a core subunit of the human MLL and SET1 histone H3K4 methyltransferase complex and is highly important for global H3K4 methylation and *HOX* gene activation in human cells. Human CHD1 preferentially binds to H3K4me3 a hallmark of actively transcribed chromatin. Recently, it was reported the CHD1 and WDR5 are a potential target for NS1 protein of influenza A H3N2 subtype possess a histone H3K4 like sequence at its CTD and adapts antiviral response [83].

The bromodomain (BRD) is a conserved structural module of chromatin-associated proteins, and histone acetyltransferases play a dynamic role in regulating chromatin-based gene transcription. BRD specifically binds to the acetylated histones and regulates gene expression [84]. Recent affinity purification-based-mass spectrometry results indicate the interaction of E protein of SARS-CoV-2 with BRD-containing proteins BRD2, BRD4 disrupting the activity of BRD histones binding by mimicking histone structure. The N terminus of histone 2A shares local sequence similarity over an alpha helix about 15 residues some of which are in the transmembrane segment of protein E, which suggests mimicking protein E’s action on histone which disrupts its interaction with BRD2, thus evading from host immune defense. Interaction analysis revealed the affinity of Nsp5 (C145A) with TRMT1 and wild-type Nsp5 with TRMT1 and HDAC2. Taking both wild-type and catalytic dead constructs (C145A) of Nsp5 of SARS-CoV-2 indicates, wild-type Nsp5 exhibits high confidence interaction with epigenetic regulator HDAC2 predicted a cleavage site between the nuclear localization sequence and HDAC domain and suggested an inhibitory effect of Nsp5 on HDAC2 transport into the nucleus. Furthermore, Nsp5 removes zinc finger and the nuclear localization signal of TRMT1 mediates mitochondrial localization (Figure 2) [83]. Fascinating results obtained by the same study from chemoinformatics data indicate valproic acid and the preclinical candidate apicidin possess HDAC2 inhibitory activity with an affinity of 5 and 120 nM, clinical compounds like ABBV-744 and CPI-0610 on BRD2 and BRD4 with affinities of 2 and 39 nM, respectively.

### Coronavirus modulates innate epigenetic signaling

The potential role of the pathogen-related receptor (PRR) and pathogen-associated molecular pattern, toll-like receptor (TLR), JAK-STAT, NF-κB signaling in orchestration against the viral pathogenesis was previously known. TLR2 mRNA was observed to increase in patients with SARS infection. Previous evidence indicates an increase in NF-κB signaling in response to the TLR2 signaling in monocytes in *in vitro* conditions [84]. Spike proteins of SARS-CoV are cleaved by cathepsin L, factor Xa and trypsin which cleaves the spike protein into S1 and S2 and permits viral entry into the cytoplasm [85]. In epithelial and fibroblast cells displaying ACE2 receptor induces IL-8 production in response to S protein via the AP-1 pathway [86]. Subsequent evidence indicates crucial roles of specific accessory proteins encoded by MERS-CoV antagonize NF-κB signaling to evade the host defense. Transport of NF-κB into the nucleus is stimulated by the destruction of IkBs, where NF-κB induces activation of cytokine genes. Upon viral infection activation of TLR and retinoic acids inducible gene like receptors (RLRs) and nucleic acid sensors recognize the pathogen-associated molecular pattern. The role of *RIG-1* and MDA-5 via MAVS in priming IkB ubiquitination by recruiting TRAF and TAK1 which induces NF-κB activation [87]. However, previous evidence indicates the viral origin of different proteins and proteases causes inactivation of these adaptor molecules leading to silencing of the NF-κB paving the way for immune evasion [88]. A recent infection experiment of coronavirus with 229E cells indicates that p65 chromatin recruitment is highly crucial in NF-κB and its target gene induction. P65 occupying regions are enhancers elements, promoter-transcription start site regions characterized by increased acetylation of H3 and H4 histones which are stimulated by the activity of transcription factor induced after CoV-2 infection and leads to expression of genes associated with antiviral response [89]. Activation of NF-κB allows the synthesis of the A20 protein required for efficient viral replication [89]. Historically, recruitment of inducible transcription factors to the enhancer region has H3K4me1 and H3K27ac and increases the acetylation of H3K36 and H4K5 in the chromatin structure near the promoter region determines virus-induced host cell response in a nuclear transcription-dependent mechanism. The possible interaction of viral protein components with different DNMTs have been well established [61,90]. The potent role of IL-32 in exerting its antiviral response is confined to its ability by inducing the pro-inflammatory cytokines and differentiation of monocytes into macrophages.

Demethylation in the CREB binding site increases the binding of CREB to the promoter followed by* IL-32* transcriptional activation in influenza A virus-infected cells. Influenza virus activates *IL-32* expression by activating NF-κB and CREB with site-specific demethylation of CRE in the *IL-32* promoter region. Inactivation of DNMT1 and DNMAT3b causes hypomethylation of *IL-32* promoter in response to influenza virus infection, indicating a host’s protective mechanism in preventing viral replication [91]. Similar findings were reported by Fang *et al.* (2012), where the downregulation of DNMT3a and DNMT3b, but not that of DNMT1, involves a COX2 dependent IFN-λ1 production by increase NF-κB signaling. The result of this study indicates increased activity of miRNA (mir29) in A549 cells and PBMC derived from the influenza patients induces PKA-mediated phosphorylation of CREB1 and inhibition of DNMTs activity and contributes to COX2 and PGE2 expression [62]. Mechanistic evidence indicates infection with influenza virus induces an increase in the expression of the host methyltransferase Setdb2, which mediates trimethylation of histone H3 Lys9 (H3K9) at the *Cxcl1* promoter and make the host susceptible to superinfection with Streptococcus pneumonia [92]. The epigenetic alterations associated with CoV-2 infection are summarized in Table 1.

**Table 1.** Epigenetic alterations associated with COVID-19 infection.

Viral components	Host machinery	Epigenetic change	Response	Ref.
Protein 3b	RUNX1b	Recruitment	T cell function cytokine response	[93,94]
Covid19	NF-κB TNF-α MCP-1	HDAC2, HDAC5	Inflammation	[95,96]
Nsp16 Nsp13 Nsp14	2′-O-MT activity 5′-triphosphatase and helicase activity N7MT activity	Methylation and mimic of Cap1 structure	Immune evasion from interferon response by protecting of viral RNA from 5′-3′ exonuclease activity	[97,98]
CoV infection	Hsp90 induced mTOR pathway	SMYD2 (Lysine methyltransferase)	Autophagy	[99,100]
CoV infection	p65 induced NF-κB activity	H3H4 acetylation	Antiviral response	
Cov Infection	BRD2	Mimics H2A histone	Host immune evasion	[83]
Nsp5	TRMT1 HDAC2	TRMT1 HDAC2	Prevents HDAC2 transport to nucleus induces mitochondrial localization of HDAC2	[83]
NL63/ IDP or IDR	Host SLiM protein	Mimics Histone H3	Immune evasion by surpassing Interferon response	[79,80]
MERS-CoV	TNF-α Interferon	H3K27me3	Antagonizes antigen presentation immune evasion	[70,75]
Spike protein	ACE2R	Methylation at CpG site	Viral entry and pathogenesis	[61]

IDR: Intrinsically disordered region; MERS-CoV: Middle East respiratory syndrome coronavirus.

## The epigenetic mechanism controls viral RNA replication

Extracellular vesicles trigger epigenetic reprogramming in the host cell. Virally induced vesicle formation can trigger the multiplicity of infection. CoV-2 Nsp-3, -4 and -6 play a fundamental role in the rearrangement of the host cell membrane and required for the establishment of replication-transcription complexes, called replication organelles, which are nothing but the double-membrane vesicles are characteristic features of all RNA viruses including CoV-2 for making stable infection in the host [101,102]. The marginalization of host cell chromatin, a proliferation of nuclear membrane are the prime events during herpes virus infection. However, such molecular events in CoV-2 vesicle are not reported. Vesicle fusion with other cellular components and chromatin disassembly in a GTPase Ran mediated manner was also described in the previous report [103].

### 5′ OMT as a molecular target plays a crucial role in evasion from the immune system

The exploitation of host synthetic machinery or encoding own proteins to counteract innate immune response is crucial for establishing a successful infection strategy. Methylation of transcriptome involving different RNAs like tRNA, mRNA, rRNA and other noncoding RNAs is crucial for regulating gene expression. Mechanistically eukaryotic mRNA is capped at 2′-O positions (or Nm where N can be any nucleotide) of the 5′-guanosine cap by methyltransferases (MTases) to distinguish endogenous self-capped RNA from exogenous nonself RNA encoded by pathogen lacking Nm. This mechanism has been well understood at the molecular level where IFIT1 interferon-induced RNA binding protein mediates this effect by preferentially binding to viral RNAs lacking 2′-O-methylation at their 5′ end and preventing RNA translation. Seminal findings suggest a wide variety of pathogens like flaviviruses, coronavirus, Japanese encephalitis virus, mouse virus, dengue virus, SARS-CoV virus and vaccinia virus adopts such strategy for the propagation of successful replication and evasion from interferon response [104]. IFIT1 has been observed to have a higher affinity for RNA lacking 2′-O methylation, IFIT1 can out-compete eIF4E or eIF4F for binding, remove cap 0 RNA from the actively translating pool [99]. Surprisingly, specific pathogens like coronaviruses encode their own viral 2′-O MTases and mimic the host’s cap1 structure by different distinct mechanisms rendered them a potential target for drug development [105]. *ORF1* translated into polyproteins (ppla and ppl1ab) which undergoes co- and post-translational modifications and forms 16 nonstructural proteins nsp 1–16. Bioinformatics study revealed SAM-dependent RNA 2′-O-MT activity of nsp16, where nsp13 function as 5′triphosphatase and helicase activity whereas nsp14 exhibits N7MT activity. This 5′ cap protects the viral RNA from degradation by altering 5′-3′ exonuclease activity and induces its translation by stimulating the preinitiation complex formation. Although the exact role of SARS-CoV-2 2′-O-MT is unknown, it plays an integral part in a viral replicase-transcriptase complex on its interactions with other viral proteins implicated in the formation of a 3′ terminal protein complex. Recent findings from computational analysis study revealed that dolutegravir and bictegravir are potential drug candidates to target SARS-CoV2 2′-O-MT activity [100].

### Coronavirus uses Hsp90-mediated epigenetic process to hijack the infected cells

Historically, histone modification plays a significant role in the epigenetic silencing of endogenous retroviruses. It was observed that ZFP809, a member of the KRAB-ZFP family, induces the silencing of endogenous retroviruses in a sequence-specific manner via recruitment of heterochromatin-inducing complexes. Epigenetic mark involving Histone 3 Lys9 trimethylation (H3K9me3) in host-associated with tightly inactive repressed chromatin [103]. ZFP809 binding to proline tRNA primer-binding site used by some retroviruses to prime reverse transcription. ZFP809 recruits the KRAB domain binding corepressor KAP1 (TRIM28, TIF1b), which induces silencing via recruitment of histone deacetylases, HP1 and the histone methyltransferase SETDB1 (ESET, KMT1E) [106]. Hsp90 chaperon activity plays a vital role in TRIM28/KAP1-mediated epigenetic silencing of endogenous retroviral elements [97]. Hsp90 in influenza virus by binding to the PB2 subunit enhances the RNA polymerase activity. In the poliovirus, Hsp90 is required for proper folding of the capsid protein. Hsp90 enables viruses to hijack the infected cells through the process of autophagy in targeting the mTOR pathway by inducing the mTOR/p70S6K pathway [98]. It has also been proposed complex interaction between estrogen, Hsp90 and lysine methyltransferases (SMYD2) plays an essential role in autophagy [107]. A similar mechanism might have to go on in SARS-CoV-2-infected patients. Remarkably, a drug repositioning study suggests geldanamycin and its derivatives as the potential candidate to target Hsp90 during COVID-19 infection [95].

## Conclusion

A viral threat to humanity represents a foremost public health concern and accounts for a significant cause of morbidity and mortality for decades. Currently, COVID-19 has raised many scientific and clinical questions. A growing body of evidence indicates an evolutionary arms race and molecular cross-talk between virus and host epigenetic landscape plays a pivotal role in encouraging the altered immune response. Another question is whether SARS-CoV2 is evolutionarily adapted to subvert the host replication, transcription and proteome program by modifying the epigenetic machinery, thus evading host immune response. The evidence summarized and discussed here clearly demonstrates that RNA viruses like SARS-CoV-2 are equipped with a molecular entity to evade from host innate immunity by altering epigenetic architecture. Although SARS-CoV-2 modifies the host phenotype by potent epigenetic types of machinery like ACE2R methylation, interfering with host replication machinery, altering antigen presentation and interferon response, innate epigenetic signaling and histone mimicry, however, the mechanistic basis of altered chromatin dynamics and viral antagonism of SARS-CoV-2 is still not thoroughly investigated. Therefore, future studies should explore and generate a more comprehensive map of important epigenetic events in the histone induced by SARS-CoV-2. Integration of the epigenetically technological approach will allow understanding of the epigenetic landscape of immune response induces by SARS-CoV-2 and prediction of a pharmacological target for therapeutic efficacy.

## Future perspective

Evidence from both MERS-CoV and SARS-CoV-2 infection indicate a significant decrease in the peripheral lymphocytes resulting in lymphopenia, thrombocytopenia, pneumonia and high CRP contributes respiratory dysfunction. Existing evidence also demonstrated the possible association of pulmonary injury with various immune cells like neutrophil and macrophage infiltration and concomitant increase in the concentration of interleukins, G-CSF, TNF-α, interferon-gamma IP10, MCP1 and MIP1A, D-dimers, alanine transaminases, lactate dehydrogenase, creatine kinase, amylase and ferritin [96,108].

Although the onset of upper acute respiratory symptoms like fever, fatigue, cough and digestive symptoms like nausea, vomiting, abdominal pain, diarrhoea is typical, however patients with severe phenotypes are characterized by pneumonia, sepsis, encephalopathy, acute respiratory distress syndrome, acute kidney injury, cardiomyopathy, pulmonary dysfunction, endothelial injury and coagulation abnormality. Considerable challenges with antimalarial drugs, antiviral agents, antiparasitic agents, immune modulators, anti-inflammatory agents, anti-angiotensin receptors and traditional herbal remedies have shown to be beneficial [109]. Moreover, till recently, no suitable US FDA-approved agent has demonstrated therapeutic efficacy in randomized clinical trials. Although cytokine storm seems crucial in modulating clinical severity, however, due to insufficient data and lack of strong evidence until recently, it is unknown whether cytokine storm is the major confounding factor modulating pathological complexity. In contrast, further emerging evidence supports the notion that the phenotype may be manifested due to endothelial dysfunction and systemic inflammatory response [93,94].

In the battle of nature and host immunity, the role of epigenetic is of tremendous importance elucidating a scientific heft to the current scenario. Although it was previously experienced that mechanistic underpinnings governing the epigenetic process are highly deterministic in affecting psychological traits like intelligence, personality, sexuality, now it is proved that epigenetics plays a significant role in modulating the infectivity and host-pathogen interaction. A host defense mechanism by manipulating epigenetic process is significant for survival and successful infection strategy by viruses. The evolution of multiple pathways played by the viruses’ structural and accessory proteins targeting the epigenetic readers, writers and erasers result in the variable of expression of proteins crucial for host defense. A viral infection is significantly associated with the exploitation of host cellular immune behavior and potent cellular signaling process. Although the immune strategy system has developed to tackle the viral attack from the perspective of the evolutionary point of view, its virus also owns the battle by developing a predominate strategy to counteract the immune system exaggerated by its adaptation and conducive environment. Epidemiological evidence indicates that viral infection and release are associated with several novel strategies induced by a virus sufficient to evade the host’s innate and adaptive immunity. Intracellular nature, low fidelity of RNA polymerase, limited genome size, resistance to physical injury make the virus ideal to manipulate the host immune system renders it to evade the immune control mechanisms [110]. Although the epigenetic mechanism controlling gene behavior is well established, understanding the primary mechanism responsible for fine-tune the epigenetic target by coronaviruses and its association with the clinical severity and disease outcome is need to be understood in detail. COVID-19 has become the primary cause of morbidity and mortality worldwide. Owing to its large extent of infectivity, lack of sufficient knowledge in understanding the pathogenesis, devoid of ideal potent therapeutic agent, vaccine and population with asymptomatic phenotype rendered it as an emerging issue in the current scenario.

It is highly crucial to understand the potential cross-talk between viruses and host; for example mechanism of viral latency, reactivation of viral latency, virus localization to different chromatin sites, recognition of potent epigenetic signature as a result of association with different stages of infection and chromatin basis of immune regulatory genes. In the past few years, significant advancement in several technological approaches has substantially contributed to understanding nuclear architecture and chromatin network. Advancement in the single cell-based chromatin analysis approach; microscopy-based single-molecule real-time imaging for chromatin dynamics, epigenome microarray, chromatin immune precipitation with next-generation sequencing, and FISH approach will be of an immense requirement to understand the dynamism of host chromatin and virus interaction. It keeps the present alarming situation in mind; there are no epigenetic drugs with clinical efficacy to control the COVID-19 infection. Therefore, the future study should focus on the detailed understanding and elucidation of the novel paradigm involved between COVID-19 and host epigenetic to develop epigenetic agents with potential efficacy.

**Executive summary**

**Overview**

- Currently, COVID-19 is the significant public health concern and exhibits a prominent cause of morbidity and mortality worldwide.
- Viruses have improved several potent mechanisms to efficiently propagate inside the host by fine-tuning the host epigenetic program leading to evasion from innate and adaptive immunity.
- Both DNA and RNA viruses regulate the epigenetic players like HAT, DNMTs, HDAC culminating into activation and repression of the specific genetic mark in the promoter of innate signaling cascade and thus subvert from host defense.

**The epigenetic perspective of SARS-CoV-2:**

***ACE2R* methylation determines SARS-CoV-2 entry into the host cell**

- *ACE2* gene methylation across three CpG sites (cg04013915, cg08559914, cg03536816) in lung epithelial cells is of paramount importance SARS-CoV-2 infection.
- SARS-CoV-2 may regulate ACE2 expression by controlling the SIRT1 and KDM5B activity.

**SARS-CoV-2 epigenetically alters antigen presentation & Interferon response**

- SARS-CoV-2 significantly delayed interferon-stimulated gene expression.
- SARS-CoV-2 may regulate Type I and III IFN response by modulating H3K27me3 and H3K4me3 histone mark.
- Monocyte and macrophage-mediated inflammatory response associated with COVID-19 are induced by acetylation and deacetylation of histones.

**Histone mimicry as a basis for modulation of gene expression & immune evasion**

- SARS-CoV-2 protein with bromodomain and protein E mimics bromodomain histones and evades from host immune response.
- However, there is a paucity of literature pertinent to this field.

**Coronavirus modulates innate epigenetic signaling**

- SARS-CoV-2 may regulate NF-κB signaling by p65 chromatin recruitment.
- However, very few works have been conducted in this field.
- SARS-CoV-2, 2′-O MTases mimics the host’s cap1 structure and plays a vital role in immune evasion.
- Coronavirus uses Hsp90-mediated epigenetic process to hijack the infected cells.

**Epigenetic tools to study the evasion strategy of SARS-CoV-2**

- Several technological approaches have contributed to the understanding of nuclear architecture and chromatin network substantially.
- Advancement in the single cell-based chromatin analysis approach; microscopy-based single-molecule real-time imaging for chromatin dynamics, epigenome microarray, chromatin immune precipitation with next-generation sequencing, will be highly beneficial to understand the dynamism of host chromatin and virus interaction.

**Conclusion & future perspective**

- Although the current review highlighted some of the critical epigenetic events associated with SARS-CoV-2 immune evasion, the detailed mechanism is yet to be elucidated.
- Future studies should focus on the novel paradigm involved between host and SARS-CoV-2 and the epigenetic basis of immune evasion to predict pharmacological targets and therapeutic interventions.

## References

[1] WHO. Coronavirus disease (COVID-19) Weekly Epidemiological Update and Weekly Operational Update. https://www.who.int/emergencies/diseases/novel-coronavirus-2019/situation-reports

[2] Lu R, Zhao X, Li J, Niu P Genomic characterization and epidemiology of 2019 novel coronavirus: implications for virus origins and receptor binding. Lancet 395(10224), 565–574 (2020). 32007145 10.1016/S0140-6736(20)30251-8 PMC7159086

[3] Zhou P, Yang XL, Wang XG A pneumonia outbreak associated with a new coronavirus of probable bat origin. Nature 579(7798), 270–273 (2020). 32015507 10.1038/s41586-020-2012-7 PMC7095418

[4] Tang X, Wu C, Li X, Song Y On the origin and continuing evolution of SARS-CoV-2. Nat. Sci. Rev. 7(6), 1012–1023 (2020). 10.1093/nsr/nwaa036 PMC7107875 34676127

[5] Huang C, Wang Y, Li X Clinical features of patients infected with 2019 novel coronavirus in Wuhan, China. Lancet 395(10223), 497–506 (2020). 31986264 10.1016/S0140-6736(20)30183-5 PMC7159299

[6] Wu JT, Leung K, Leung GM. Now casting and forecasting the potential domestic and international spread of the 2019-nCoV outbreak originating in Wuhan, China: a modelling study. Lancet 395(10225), 689–697 (2020). 32014114 10.1016/S0140-6736(20)30260-9 PMC7159271

[7] Lu F, Tsai K, Chen HS Identification of host-chromosome binding sites and candidate gene targets for Kaposi's sarcoma-associated herpesvirus LANA. J. Vir. 86(10), 5752–5762 (2012). 10.1128/JVI.07216-11 PMC3347294 22419807

[8] Obata Y, Furusawa Y, Hase K. Epigenetic modifications of the immune system in health and disease. Imm. Cell Biol. 93(3), 226–232 (2015). 10.1038/icb.2014.114 25666097

[9] Sato A, Saito Y, Sugiyama K Suppressive effect of the histone deacetylase inhibitor suberoylanilide hydroxamic acid (SAHA) on hepatitis C virus replication. J. Cell. Biochem. 114(9), 1987–1996 (2013). 23519646 10.1002/jcb.24541

[10] Tzika E, Dreker T, Imhof A. Epigenetics and metabolism in health and disease. Front. Gen. 9, 361 (2018). 10.3389/fgene.2018.00361 PMC6153363 30279699

[11] Marcos-Villar L, Díaz-Colunga J, Sandoval J Epigenetic control of influenza virus: role of H3K79 methylation in interferon-induced antiviral response. Sci. Rep. 8(1), 1–13 (2018). 29352168 10.1038/s41598-018-19370-6 PMC5775356

[12] Shamay M, Krithivas A, Zhang J Recruitment of the *de novo* DNA methyltransferase Dnmt3a by Kaposi's sarcoma-associated herpesvirus LANA. Proc. Natl Acad. Sci. 103(39), 14554–14559 (2006). 16983096 10.1073/pnas.0604469103 PMC1599998

[13] Di Bartolo DL, Cannon M, Liu YF KSHV LANA inhibits TGF-β signalling through epigenetic silencing of the TGF-β Type II receptor. Blood 111(9), 4731–4740 (2008). 18199825 10.1182/blood-2007-09-110544 PMC2343603

[14] Rossetto CC, Pari GS. Kaposi's sarcoma-associated herpesvirus non-coding polyadenylated nuclear RNA interacts with virus-and host cell-encoded proteins and suppresses expression of genes involved in immune modulation. J. Virol. 85(24), 13290–13297 (2011). 21957289 10.1128/JVI.05886-11 PMC3233155

[15] Paschos K, Smith P, Anderton E Epstein-barr virus latency in B cells leads to epigenetic repression and CpG methylation of the tumour suppressor gene Bim. PLoS Pathog. 5(6), e1000492 (2009). 19557159 10.1371/journal.ppat.1000492 PMC2695769

[16] Hino R, Uozaki H, Murakami N Activation of DNA methyltransferase 1 by EBV latent membrane protein 2A leads to promoter hypermethylation of PTEN gene in gastric carcinoma. Can. Res. 69(7), 2766–2774 (2009). 10.1158/0008-5472.CAN-08-3070 19339266

[17] Okamoto Y, Shinjo K, Shimizu Y Hepatitis virus infection affects DNA methylation in mice with humanized livers. Gastroenterol. 146(2), 562–572 (2014). 10.1053/j.gastro.2013.10.056 24184133

[18] Jain S, Chang TT, Chen S Comprehensive DNA methylation analysis of hepatitis B virus genome in infected liver tissues. Sci.Rep. 5(1), 1–11 (2015). 10.1038/srep10478 PMC4650678 26000761

[19] Schinzari V, Barnaba V, Piconese S. Chronic hepatitis B virus and hepatitis C virus infections and cancer: synergy between viral and host factors. Clin. Microbiol. Infect. 21(11), 969–974 (2015). 26163104 10.1016/j.cmi.2015.06.026

[20] Zheng DL, Zhang L, Cheng N Epigenetic modification induced by hepatitis B virus X protein via interaction with *de novo* DNA methyltransferase DNMT3A. J. Hepat. 50(2), 377–387 (2009). 10.1016/j.jhep.2008.10.019 19070387

[21] Reeder JE, Kwak YT, McNamara RP HIV Tat controls RNA Polymerase II and the epigenetic landscape to transcriptionally reprogram target immune cells. Elife 4, e08955 (2015). 26488441 10.7554/eLife.08955 PMC4733046

[22] Pion M, Jaramillo-Ruiz D, Martínez A HIV infection of human regulatory T cells downregulates Foxp3 expression by increasing DNMT3b levels and DNA methylation in the FOXP3 gene. AIDS 27(13), 2019–2029 (2013). 24201117 10.1097/QAD.0b013e32836253fd

[23] Nitzsche A, Paulus C, Nevels M. Temporal dynamics of cytomegalovirus chromatin assembly in productively infected human cells. J. Virol. 82(22), 11167–11180 (2008). 18786996 10.1128/JVI.01218-08 PMC2573275

[24] Levine AJ. The common mechanisms of transformation by the small DNA tumor viruses: the inactivation of tumor suppressor gene products: p53. Virology 384(2), 285–293 (2009). 19081592 10.1016/j.virol.2008.09.034

[25] Lynch KL, Gooding LR, Garnett-Benson C Epigenetics and the dynamics of chromatin during adenovirus infections. FEBS Lett. 593(24), 3551–3570 (2019). 31769503 10.1002/1873-3468.13697 PMC6938402

[26] Fonseca GJ, Thillainadesan G, Yousef AF Adenovirus evasion of interferon-mediated innate immunity by direct antagonism of a cellular histone post-translational modification. Cell Host Microbe 11(6), 597–606 (2012). 22704620 10.1016/j.chom.2012.05.005

[27] Bauman Y, Nachmani D, Vitenshtein A An identical miRNA of the human JC and BK polyomaviruses targets the stress-induced ligand ULBP3 to escape immune elimination. Cell Host Microbe 9(2), 93–102 (2011). 21320692 10.1016/j.chom.2011.01.008

[28] Liu Y, Sun J, Zhang H Ebola virus encodes a miR-155 analog to regulate importin-α5 expression. Cell. Mol. Life Sci. 73(19), 3733–3744 (2016). 27094387 10.1007/s00018-016-2215-0 PMC11108478

[29] Sullivan CS, Grundhoff AT, Tevethia S SV40-encoded microRNAs regulate viral gene expression and reduce susceptibility to cytotoxic T cells. Nature 435(7042), 682–686 (2005). 15931223 10.1038/nature03576

[30] Naqvi AR, Shango J, Seal A Viral miRNAs alter host cell miRNA profiles and modulate innate immune responses. Front. Immunol. 6(9), 433 (2018). 10.3389/fimmu.2018.00433 PMC5845630 29559974

[31] Seo GJ, Chen CJ, Sullivan CS. Merkel cell polyomavirus encodes a microRNA with the ability to autoregulate viral gene expression. Virology 383(2), 183–187 (2009). 19046593 10.1016/j.virol.2008.11.001

[32] Nachmani D, Stern-Ginossar N, Sarid R Diverse herpesvirus microRNAs target the stress-induced immune ligand MICB to escape recognition by natural killer cells. Cell Host Microbe 5(4), 376–385 (2009). 19380116 10.1016/j.chom.2009.03.003

[33] Wu S, He L, Li Y miR-146a facilitates replication of dengue virus by dampening interferon induction by targeting TRAF6. J. Infect. 67(4), 329–341 (2013). 23685241 10.1016/j.jinf.2013.05.003

[34] Shirakawa K, Chavez L, Hakre S Reactivation of latent HIV by histone deacetylase inhibitors. Trends Microbiol. 21(6), 277–285 (2013). 23517573 10.1016/j.tim.2013.02.005 PMC3685471

[35] Perrine SP, Hermine O, Small T A Phase I/II trial of arginine butyrate and ganciclovir in patients with Epstein-Barr virus-associated lymphoid malignancies. Blood 109(6), 2571–2578 (2007). 17119113 10.1182/blood-2006-01-024703 PMC1852196

[36] Ghosh SK, Perrine SP, Williams RM Histone deacetylase inhibitors are potent inducers of gene expression in latent EBV and sensitize lymphoma cells to nucleoside antiviral agents. Blood 119(4), 1008–1017 (2012). 22160379 10.1182/blood-2011-06-362434 PMC3271713

[37] Chan AT, Tao Q, Robertson KD Azacitidine induces demethylation of the Epstein-Barr virus genome in tumors. J. Clin. Oncol. 22(8), 1373–1381 (2004). 15007085 10.1200/JCO.2004.04.185

[38] Thiagarajan D, Dev RR, Khosla S. The DNA methyltransferase Dnmt2 participates in RNA processing during cellular stress. Epigenetics 6(1), 103–113 (2011). 20864816 10.4161/epi.6.1.13418

[39] Samantarrai D, Dash S, Chhetri B Mallick. Genomic and epigenomic cross-talks in the regulatory landscape of miRNAs in breast cancer. Mol. Can. Res. 11(4), 315–328 (2013). 10.1158/1541-7786.MCR-12-0649 23360796

[40] Upadhyay M, Sharma N, Vivekanandan P. Systematic CpT (ApG) depletion and CpG excess are unique genomic signatures of large DNA viruses infecting invertebrates. PLoS ONE 9(11), e111793 (2014). 25369195 10.1371/journal.pone.0111793 PMC4219779

[41] Choudhury JH, Ghosh SK. Promoter hypermethylation profiling identifies subtypes of head and neck cancer with distinct viral, environmental, genetic and survival characteristics. PLoS ONE 10(6), e0129808 (2015). 26098903 10.1371/journal.pone.0129808 PMC4476679

[42] Bazot Q, Paschos K, Skalska L et al.. Epstein-Barr virus proteins EBNA3A and EBNA3C together induce expression of the oncogenic microRNA cluster miR-221/miR-222 and ablate expression of its target p57KIP2.. PLoS Pathog. 11(7), e1005031 (2015). 26153983 10.1371/journal.ppat.1005031 PMC4496050

[43] Manocha GD, Mishra R,Sharma N et al.. Regulatory role of TRIM21 in the type-I interferon pathway in Japanese encephalitis virus-infected human microglial cells.. J. Neuroinflammation 11(1),1-12 (2014). 24485101 10.1186/1742-2094-11-24 PMC3922089

[44] Bhattacharjee S, Bose P, Patel K Transcriptional and epigenetic modulation of autophagy promotes EBV oncoprotein EBNA3C induced B-cell survival. Cell Death Dis. 9(6), 1–18 (2018). 29789559 10.1038/s41419-018-0668-9 PMC5964191

[45] Rastogi M, Singh SK. Modulation of type-I interferon response by hsa-miR-374b-5p during Japanese encephalitis virus infection in human microglial cells. Front. Cell. Infect. Microbiol. 9, 291 (2019). 31448245 10.3389/fcimb.2019.00291 PMC6695837

[46] Chattopadhyay D, Mukhopadhyay A, Ojha D Immuno-metabolic changes in herpes virus infection. Cytokine 112, 52–62 (2018). 29960669 10.1016/j.cyto.2018.06.028

[47] Hoffmann M, Kleine-Weber H, Schroeder S SARS-CoV-2 cell entry depends on ACE2 and TMPRSS2 and is blocked by a clinically proven protease inhibitor. Cell 181(2), 271–280 (2020). 32142651 10.1016/j.cell.2020.02.052 PMC7102627

[48] Yan T, Xiao R, Lin G. Angiotensin-converting enzyme 2 in severe acute respiratory syndrome coronavirus and SARS-CoV-2: a double-edged sword? FASEB J. 34(5), 6017–6026 (2020). 32306452 10.1096/fj.202000782 PMC7264803

[49] Mudersbach T, Siuda D, Kohlstedt K Epigenetic control of the angiotensin-converting enzyme in endothelial cells during inflammation. PLoS ONE 14(5), e0216218 (2019). 31042763 10.1371/journal.pone.0216218 PMC6494048

[50] Zhang XH, Zeng ZP, Li HZ Expression of renin-angiotensin-aldosterone system in human adipose tissues. Zhongguo Yi Xue ke Xue yuan xue bao. Acta Academiae Medicinae Sinicae 28(6), 766 (2006). 17260463

[51] Corley MJ, Ndhlovu LC. DNA methylation analysis of the COVID-19 host cell receptor, angiotensin I converting enzyme 2 gene (ACE2) in the respiratory system reveal age and gender differences. (2020).

[52] Joubert BR, Felix JF, Yousefi P DNA methylation in newborns and maternal smoking in pregnancy: genome-wide consortium meta-analysis. Am. J. Hum. Genet. 98(4), 680–696 (2016). 27040690 10.1016/j.ajhg.2016.02.019 PMC4833289

[53] Stueve TR, Li WQ, Shi J Epigenome-wide analysis of DNA methylation in lung tissue shows concordance with blood studies and identifies tobacco smoke-inducible enhancers. Hum. Mol. Genet. 26(15), 3014–3027 (2017). 28854564 10.1093/hmg/ddx188 PMC5886283

[54] Zhao Y, Zhao Z, Wang Y Single-cell RNA expression profiling of ACE2, the receptor of SARS-CoV-2. BioRxiv 202(5), 756–759 (2020). 10.1164/rccm.202001-0179LE PMC7462411 32663409

[55] Sawalha AH, Zhao M, Coit P Epigenetic dysregulation of ACE2 and interferon-regulated genes might suggest increased COVID-19 susceptibility and severity in lupus patients. Clin. Immunol. 6(215), 108410 (2020). 10.1016/j.clim.2020.108410 PMC7139239 32276140

[56] Clarke NE, Belyaev ND, Lambert DW Epigenetic regulation of angiotensin-converting enzyme 2 (ACE2) by SIRT1 under conditions of cell energy stress. Clin. Sci. 126(7), 507–516 (2014). 10.1042/CS20130291 24147777

[57] Ansari A, Arya R, Sachan S Immune memory in mild COVID-19 patients and unexposed donors from India reveals persistent T cell responses after SARS-CoV-2 infection. medRxiv (2020). 10.3389/fimmu.2021.636768 PMC7991090 33777028

[58] Pinto BG, Oliveira AE, Singh Y ACE2 expression is increased in the lungs of patients with comorbidities associated with severe COVID-19. MedRxiv 222(4), 556–563 (2020). 10.1093/infdis/jiaa332 PMC7377288 32526012

[59] Channappanavar R, Zhao J, Perlman S. T cell-mediated immune response to respiratory coronaviruses. Immunol. Res. 59(1–3), 118–128 (2014). 24845462 10.1007/s12026-014-8534-z PMC4125530

[60] Yip MS, Leung NH, Cheung CY Antibody-dependent infection of human macrophages by severe acute respiratory syndrome coronavirus. Virol. J. 11(1), 1–11 (2014). 24885320 10.1186/1743-422X-11-82 PMC4018502

[61] Menachery VD, Eisfeld AJ, Schäfer A Pathogenic influenza viruses and coronaviruses utilize similar and contrasting approaches to control interferon-stimulated gene responses. MBio 5(3), e01174–14 (2014). 24846384 10.1128/mBio.01174-14 PMC4030454

[62] Fang J, Hao Q, Liu L Epigenetic changes mediated by microRNA miR29 activate cyclooxygenase 2 and lambda-1 interferon production during viral infection. J. Virol. 86(2), 1010–1020 (2012). 22072783 10.1128/JVI.06169-11 PMC3255816

[63] Marcos-Villar L, Nieto A. The DOT1L inhibitor pinometostat decreases the host-response against infections: considerations about its use in human therapy. Sci. Rep. 9, 9 (2019). 31727944 10.1038/s41598-019-53239-6 PMC6856118

[64] Ishii M, Wen H, Corsa CA Epigenetic regulation of the alternatively activated macrophage phenotype. Blood 114(15), 3244–3254 (2009). 19567879 10.1182/blood-2009-04-217620 PMC2759649

[65] Kramer JM, Kochinke K, Oortveld MA Epigenetic regulation of learning and memory by *Drosophila* EHMT/G9a. PLoS Biol. 9(1), e1000569 (2011). 21245904 10.1371/journal.pbio.1000569 PMC3014924

[66] Menachery VD, Schäfer A, Burnum-Johnson KE MERS-CoV and H5N1 influenza virus antagonize antigen presentation by altering the epigenetic landscape. Proc. Natl Acad. Sci. 115(5), E1012–E1021 (2018). 29339515 10.1073/pnas.1706928115 PMC5798318

[67] Quintin J, Saeed S, Martens JH Candida albicans infection affords protection against reinfection via functional reprogramming of monocytes. Cell Host Microbe 12(2), 223–232 (2012). 22901542 10.1016/j.chom.2012.06.006 PMC3864037

[68] Liu S, Liu L, Xu G Epigenetic modification is regulated by the interaction of influenza A virus non-structural protein 1 with the *de novo* DNA methyltransferase DNMT3B and subsequent transport to the cytoplasm for K48-linked polyubiquitination. J. Virol. 93(7), e01587–18 (2019). 30651365 10.1128/JVI.01587-18 PMC6430541

[69] Eastman AJ, Xu J, Bermik J, Potchen N Epigenetic stabilization of DC and DC precursor classical activation by TNFα contributes to protective T cell polarization. Sci. Advan. 5(12), eaaw9051 (2019). 31840058 10.1126/sciadv.aaw9051 PMC6892624

[70] Weiss U, Möller M, Husseini SA Inhibition of HDAC Enzymes Contributes to Differential Expression of Pro-Inflammatory Proteins in the TLR-4 Signaling Cascade. Int. J. Mol. Sci. 21(23), 8943 (2020). 10.3390/ijms21238943 PMC7728096 33255670

[71] Leoni F, Zaliani A, Bertolini G The antitumor histone deacetylase inhibitor suberoylanilide hydroxamic acid exhibits anti-inflammatory properties via suppression of cytokines. Proc. Natl Acad. Sci. 99(5), 2995–3000 (2002). 11867742 10.1073/pnas.052702999 PMC122461

[72] Ito K, Hanazawa T, Tomita K Oxidative stress reduces histone deacetylase 2 activity and enhances IL-8 gene expression: role of tyrosine nitration. Biochem. Biophy. Res. Comm. 315(1), 240–245 (2004). 10.1016/j.bbrc.2004.01.046 15013452

[73] Drexhage RC, Knijff EM, Padmos RC The mononuclear phagocyte system and its cytokine inflammatory networks in schizophrenia and bipolar disorder. Expert Rev. Neurotherapeut. 10(1), 59–76 (2010). 10.1586/ern.09.144 20021321

[74] Bristow CA, Shore P. Transcriptional regulation of the human MIP-1α promoter by RUNX1 and MOZ. Nucleic Acids Res. 31(11), 2735–2744 (2003). 12771199 10.1093/nar/gkg401 PMC156734

[75] Law HK, Cheung CY, Ng HY Chemokine up-regulation in sars-coronavirus–infected, monocyte-derived human dendritic cells. Blood 106(7), 2366–2374 (2005). 15860669 10.1182/blood-2004-10-4166 PMC1895271

[76] Varshney B, Agnihotram S, Tan YJ SARS coronavirus 3b accessory protein modulates transcriptional activity of RUNX1b. PLoS ONE 7(1), e29542 (2012). 22253733 10.1371/journal.pone.0029542 PMC3257236

[77] Taniuchi I, Sunshine MJ, Festenstein R Evidence for distinct CD4 silencer functions at different stages of thymocyte differentiation. Mol. Cell 10(5), 1083–1096 (2002). 12453416 10.1016/s1097-2765(02)00735-9

[78] Xue B, Dunker AK, Uversky VN. Orderly order in protein intrinsic disorder distribution: disorder in 3500 proteomes from viruses and the three domains of life. J. Biomol. Str. Dynam. 30(2), 137–149 (2012). 10.1080/07391102.2012.675145 22702725

[79] Pyrc K, Berkhout B, Van Der Hoek L. The novel human coronaviruses NL63 and HKU1. J. Virol. 81(7), 3051–3057 (2007). 17079323 10.1128/JVI.01466-06 PMC1866027

[80] Marazzi I, Ho JS, Kim J Suppression of the antiviral response by an influenza histone mimic. Nature 483(7390), 428–433 (2012). 22419161 10.1038/nature10892 PMC3598589

[81] Bao Y, Bolotov P, Dernovoy D The influenza virus resource at the National Center for Biotechnology Information. J. Virol. 82(2), 596–601 17942553 10.1128/JVI.02005-07 PMC2224563

[82] Golebiewski L, Liu H, Javier RT The avian influenza virus NS1 ESEV PDZ binding motif associates with Dlg1 and Scribble to disrupt cellular tight junctions. J. Virol. 85(20), 10639–10648 (2011). 21849460 10.1128/JVI.05070-11 PMC3187509

[83] Gordon D, Jang GM, Bouhaddou M A SARS-CoV-2-Human Protein-Protein Interaction Map Reveals Drug Targets and Potential Drug Repurposing. Nature 583(7816), 459–468 (2020). 32353859 10.1038/s41586-020-2286-9 PMC7431030

[84] Josling GA, Selvarajah SA, Petter M The role of bromodomain proteins in regulating gene expression. Genes 3(2), 320–343 (2012). 24704920 10.3390/genes3020320 PMC3899951

[85] Dosch SF, Mahajan SD, Collins AR. SARS coronavirus spike protein-induced innate immune response occurs via activation of the NF-κB pathway in human monocyte macrophages *in vitro*. Virus Res. 142(1–2), 19–27 (2009). 19185596 10.1016/j.virusres.2009.01.005 PMC2699111

[86] Chang YJ, Liu CY, Chiang BL Induction of IL-8 release in lung cells via activator protein-1 by recombinant baculovirus displaying severe acute respiratory syndrome-coronavirus spike proteins: identification of two functional regions. J. Immunol. 173(12), 7602–7614 (2004). 15585888 10.4049/jimmunol.173.12.7602

[87] Lee JY, Bae S, Myoung J. Middle East respiratory syndrome coronavirus-encoded accessory proteins impair MDA5-and TBK1-mediated activation of NF-κB. J. Microbiol. Biotechnol. 29(8), 1316–1323 (2019). 31434175 10.4014/jmb.1908.08004

[88] Deng L, Zeng Q, Wang M Suppression of NF-κB activity: a viral immune evasion mechanism. Viruses 10(8), 409 (2018). 10.3390/v10080409 PMC6115930 30081579

[89] Poppe M, Wittig S, Jurida L NF-κB-dependent and-independent transcriptome and chromatin landscapes of human coronavirus 229E-infected cells. PLoS Pathog. 13(3), e1006286 (2017). 28355270 10.1371/journal.ppat.1006286 PMC5386326

[90] Zheng DL, Zhang L, Cheng N Epigenetic modification induced by hepatitis B virus X protein via interaction with *de novo* DNA methyltransferase DNMT3A. J. Hepat. 50(2), 377–387 (2009). 10.1016/j.jhep.2008.10.019 19070387

[91] Li W, Sun W, Liu L IL-32: a host pro-inflammatory factor against influenza viral replication is up-regulated by aberrant epigenetic modifications during influenza A virus infection. J. Immunol. 185(9), 5056–5065 (2010). 20889550 10.4049/jimmunol.0902667

[92] Schliehe C, Flynn EK, Vilagos B The methyltransferase Setdb2 mediates virus-induced susceptibility to bacterial superinfection. Nature Immunol. 16(1), 67–74 (2015). 25419628 10.1038/ni.3046 PMC4320687

[93] Kox M, Waalders NJ, Kooistra EJ Cytokine levels in critically ill patients with COVID-19 and other conditions. JAMA 324(15), 1565–1567 (2020). 10.1001/jama.2020.17052 PMC7489366 32880615

[94] Sinha P, Matthay MA, Calfee CS. Is a “cytokine storm” relevant to COVID-19? JAMA Int. Med. 180(9), 1152–1154 (2020). 10.1001/jamainternmed.2020.3313 32602883

[95] Sultan I, Howard S, Tbakhi A. Drug repositioning suggests a role for the heat shock protein 90 inhibitor geldanamycin in treating COVID-19 infection. (2020).

[96] Yuki K, Fujiogi M, Koutsogiannaki S. COVID-19 pathophysiology: a review. Clin. Immunol. (2020). 10.1016/j.clim.2020.108427 PMC7169933 32325252

[97] Hummel B, Hansen EC, Yoveva A The evolutionary capacitor HSP90 buffers the regulatory effects of mammalian endogenous retroviruses. Nature Struct. Mol. Biol. 24(3), 234 (2017). 28134929 10.1038/nsmb.3368

[98] Liu G, Zhong M, Guo C Autophagy is involved in regulating influenza A virus RNA and protein synthesis associated with both modulation of Hsp90 induction and mTOR/p70S6K signalling pathway. Int. J. Biochem. Cell Biol. 72, 100–108 (2016). 26794463 10.1016/j.biocel.2016.01.012

[99] Habjan M, Hubel P, Lacerda L Sequestration by IFIT1 impairs translation of 2′ O-unmethylated capped RNA. PLoS Pathog. 9(10), e1003663 (2013). 24098121 10.1371/journal.ppat.1003663 PMC3789756

[100] Khan RJ, Jha RK, Amera GM Targeting SARS-CoV-2: a systematic drug repurposing approach to identify promising inhibitors against 3C-like proteinase and 2′-O-ribose methyltransferase. J. Biomol. Struct. Dynam. 1–14 (2020). 10.1080/07391102.2020.1753577 PMC7189412 32266873

[101] Angelini MM, Akhlaghpour M, Neuman BW Severe acute respiratory syndrome coronavirus non-structural proteins 3, 4, and 6 induce double-membrane vesicles. MBio 4(4), (2013). 10.1128/mBio.00524-13 PMC3747587 23943763

[102] Doyle N, Hawes PC, Simpson J The porcine Deltacoronavirus replication organelle comprises double-membrane vesicles and zippered endoplasmic reticulum with double-membrane spherules. Viruses 11(11), 1030 (2019). 10.3390/v11111030 PMC6893519 31694296

[103] Prunuske AJ, Ullman KS. The nuclear envelope: form and reformation. Curr. Opin. Cell Biol. 18(1), 108–116 (2006). 16364623 10.1016/j.ceb.2005.12.004 PMC4339063

[104] Miorin L, Maestre AM, Fernandez-Sesma A Antagonism of Type I interferon by flaviviruses. Biochemi. Biophy. Res. Comm. 492(4), 587–596 (2017). 10.1016/j.bbrc.2017.05.146 PMC5626595 28576494

[105] Li SH, Dong H, Li XF Rational design of a flavivirus vaccine by abolishing viral RNA 2′-O methylation. J. Virol. 87(10), 5812–5819 (2013). 23487465 10.1128/JVI.02806-12 PMC3648161

[106] Wolf G, Yang P, Füchtbauer AC The KRAB zinc finger protein ZFP809 is required to initiate epigenetic silencing of endogenous retroviruses. Genes & development 29(5), 538–554 (2015). 25737282 10.1101/gad.252767.114 PMC4358406

[107] Obermann WM. A motif in HSP90 and P23 that links molecular chaperones to efficient estrogen receptor α methylation by the lysine methyltransferase SMYD2. J. Biol. Chem. 293(42), 16479–16487 (2018). 30190324 10.1074/jbc.RA118.003578 PMC6200951

[108] Zhou Y, Fu B, Zheng X Pathogenic T-cells and inflammatory monocytes incite inflammatory storms in severe COVID-19 patients. Nat. Sci. Rev. 7(6), 998-1002(2020). 10.1093/nsr/nwaa041 PMC7108005 34676125

[109] Hassan SA, Sheikh FN, Jamal S Coronavirus (COVID-19): a review of clinical features, diagnosis, and treatment. Cureus 12(3), e7355 (2020). 32328367 10.7759/cureus.7355 PMC7170025

[110] Hilleman MR. Strategies and mechanisms for host and pathogen survival in acute and persistent viral infections. Proc. Natl Acad. Sci. 5(101), 14560–14566 (2004). 10.1073/pnas.0404758101 PMC521982 15297608

